# Drug Target Identification with Machine Learning: How to Choose Negative Examples

**DOI:** 10.3390/ijms22105118

**Published:** 2021-05-12

**Authors:** Matthieu Najm, Chloé-Agathe Azencott, Benoit Playe, Véronique Stoven

**Affiliations:** 1 Center for Computational Biology, Mines ParisTech, PSL Research University, 75006 Paris, France; chloe-agathe.azencott@mines-paristech.fr (C.-A.A.); playe.benoit@gmail.com (B.P.); veronique.stoven@mines-paristech.fr (V.S.); 2 Institut Curie, 75248 Paris, France; 3 INSERM U900, 75428 Paris, France

**Keywords:** chemogenomic, drug discovery, target identification, false positive predictions, negative examples, machine learning, support vector machines, random forests, learning bias

## Abstract

Identification of the protein targets of hit molecules is essential in the drug discovery process. Target prediction with machine learning algorithms can help accelerate this search, limiting the number of required experiments. However, Drug-Target Interactions databases used for training present high statistical bias, leading to a high number of false positives, thus increasing time and cost of experimental validation campaigns. To minimize the number of false positives among predicted targets, we propose a new scheme for choosing negative examples, so that each protein and each drug appears an equal number of times in positive and negative examples. We artificially reproduce the process of target identification for three specific drugs, and more globally for 200 approved drugs. For the detailed three drug examples, and for the larger set of 200 drugs, training with the proposed scheme for the choice of negative examples improved target prediction results: the average number of false positives among the top ranked predicted targets decreased, and overall, the rank of the true targets was improved.Our method corrects databases’ statistical bias and reduces the number of false positive predictions, and therefore the number of useless experiments potentially undertaken.

## 1. Introduction

Drug discovery often relies on the identification of a therapeutic target, usually a protein playing a role in a disease. Then, small molecular drugs that interact with the protein target to alter disease development are designed or searched for among large molecular databases. However, there has been a renewed interest in recent years for phenotypic drug discovery, which does not rely on prior knowledge of the target. In particular, the pharmaceutical industry has invested more efforts in poorly understood rare diseases, and for which therapeutic targets have not been discovered yet. While phenotypic drug discovery has made possible the identification of a few first-in class drugs [1], once a phenotypic hit is identified, not knowing its mechanism of action is a strong limitation to fill the gap between the hit and a drug that can reach the market [2]. More fundamentally, the target points at key biological pathways involved in the disease, helping to better understand its molecular basis.

Our work aims at helping determination of the protein targets for hit molecules discovered in phenotypic screens. Identification of a drug target based solely on experiments is out of reach because it would require to design biological assays for all possible proteins. In that context, in silico approaches can reduce number of experimental tests by focusing on a limited number of high probable protein targets. Among them, Quantitative Structure-Activity Relationship (QSAR) methods were developed for that purpose [3]. They are efficient methods for the inverse problem of finding new molecules against a given target, when ligands are already known for this target. However, using them to identify the targets of a given molecule would require training a model for each protein across the protein space, which is not possible because many proteins have only few, or even no, known ligand.

Docking approaches can address this question [4], but they are restricted to proteins with known 3D structures, which is far from covering the human proteome.

In the present paper, we tackle target identification in the form of Drug-Target Interaction (DTI) prediction based on machine learning (ML) chemogenomic algorithms [5]. These approaches can be viewed as an attempt to complete a large matrix of binary interactions relating molecules to proteins (1 if the protein and molecule interact, 0 otherwise). This matrix is partially filled with known interactions reported in the literature and gathered in large databases such as the PubChem database at NCBI [6]. They can be used to train ML chemogenomic algorithms by formulating the problem of DTIs prediction as a binary classification task, where the goal is predict the probability for pairs (m,p) of molecules and proteins to interact. They can be used both to predict drugs against protein targets, or protein targets for a drug, the latter being relevant to our topic.

Various ML algorithms have been proposed for DTI predictions. They include similarity-based (or kernel-based) methods such as kernel ridge linear regression, Support Vector Machines [7], or Neighborhood Regularized Logistic Matrix Factorization (NRLMF) that decompose the interaction matrix into the product of two matrices of lower ranks that operate in two latent spaces of proteins and molecules [8]. Other ML algorithms are featured-based, which means that they rely on explicit descriptors for molecules and proteins, such as Random Forests (RF) [9], or Sparse Canonical Correspondence Analysis (SCCA) [10]. Their prediction performances are usually very high when the training data are not too “far” from the (m,p) pairs in the test set [11]. Deep learning approaches relying on protein and molecule descriptors have also been proposed, but their prediction performances outperforms those of shallow learning methods only when the training data are very abundant, or when various heterogeneous sources of information are used in the context of transfer learning [12].

However, whatever the algorithm used, training a good ML chemogenomic model is hindered by biases in the DTI databases, such as whether the molecule for which one wishes to make predictions has known interactions or not [13]. An additional issue arises when the databases only contain positive examples of (m,p) pairs known to interact, but no negative examples of (m,p) known not to interact. In this context, it is classical to assume that most unlabeled interactions are negatives, and to randomly sample negative examples among them [11]. In this work, we explore how to best choose negative examples to correct the statistical bias of databases, and reduce the number of false positive predictions, which is essential to reduce the number of biological experiments required for validation of the true protein targets. While the goal of the present paper was not to compare the prediction performances of various ML algorithms, we first compared the performances of two algorithms, namely SVM and RF, on the DrugBank dataset considered in the present study. We found that overall, SVM displayed the best results, and therefore, this algorithm was further kept to study how to correct learning bias.

## 2. Materials and Methods

### 2.1. Datasets

ML algorithms for DTI predictions need to be trained on datasets of known DTIs in which proteins and molecules are similar to those for which predictions will be performed. Hit molecules in phenotypic screens for drug discovery are mostly drug-like molecules [14], and proteins will be human proteins. We used the DrugBank database (version 5.1.5) [15] to build our training dataset, because although much smaller than other databases like PubChem or ChEMBL, it provides high quality bio-activity information regarding approved and experimental drugs, including their targets, and contains around 17,000 curated Drug-Target Interactions (DTIs). Therefore, we built a dataset called DB-Database hereafter, that comprises all (m,p) DTIs reported in DrugBank involving a human protein and a small molecular drug. Overall, the DB-Database comprises 14,637 interactions between 2670 human proteins and 5070 drug-like molecules, which make up our positive DTIs. Because training a ML algorithm also requires negative examples, we added an equal number of negative DTIs to the DB-Database following two strategies:

- Random sampling: Negative examples were randomly chosen among the pairs (m,p) that are not labeled as a DTI but such that both *m* and *p* are in the DB-Database, under the assumption that most of the unlabeled interactions are expected to be negative. This process was repeated 5 times, leading to 5 training datasets called RN-datasets (for Random Negatives-datasets) hereafter, differing only by their negative examples.
- Balanced sampling: To avoid biasing our algorithms towards proteins with many interactions, negative examples were randomly chosen among unlabeled DTIs, although in such a way that each protein and each drug appeared an equal number of times in positive and negative interactions. This process was also repeated 5 times, leading to 5 training datasets again differing only by their negative examples called hereafter BN-datasets (for Balanced Negatives-datasets). Building this set of negative DTIs is not trivial, and we propose the following algorithm:
  1. Each protein and molecule in the DB-Database has a counter corresponding initially to its number of known ligands or targets, respectively;
  2. For each protein, starting from those with the highest counter to those with a counter equal to 1, molecules are randomly chosen among those not known to interact with this protein and whose counter is greater or equal to 1;
  3. Each time a negative DTI is chosen, the counter of the corresponding protein and of the molecule is decreased by one unit;
  4. The process is repeated until all proteins and molecules counters are equal to 0.

**Figure 1:**
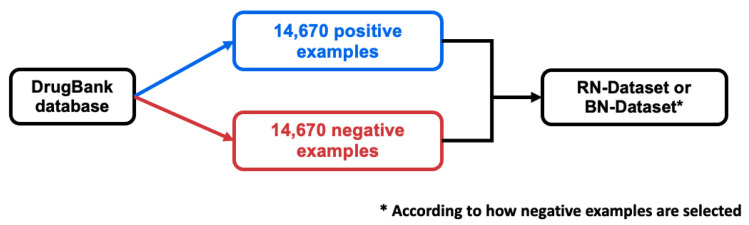
Method for building one RN-dataset (or one BN-dataset).

Overall, the RN-datasets and the BN-datasets share the same set of positive DTIs, which are those in the DB-Database, and their total number of negative DTIs are the same and equal to that of positive DTIs. The construction of one RN-dataset (or one BN-dataset) is summarized in Figure 1.

Finally, to compare the performance of the algorithm trained on the RN-datasets or the BN-datasets when predicting targets for “difficult” molecules (hit molecules will generally be “difficult” molecules, in the sense that they will have no or few known targets), we considered a small dataset of DTIs involving 200 drugs that have few known targets. We built this dataset as follows: from the 5070 molecules in the DB-Database, we kept approved drugs that do not have more than 4 targets. This leads to 560 drugs involved in 851 interactions, among which we randomly selected 200 of these positive DTIs, involving 200 different drugs, defining the so-called 200-positive-dataset. 200 negative DTIs were also randomly chosen among all unlabeled DTIs involving theses drugs and not belonging to the training RN- or BN-datasets, defining the so-called 200-negative-dataset.

All datasets are provided in the github repository mentioned under “Data Availability Statement”.

### 2.2. Machine Learning Algorithms

Throughout the paper, the main algorithm we use to address target identification through a chemogenomics approach for DTI prediction is based on the Support Vector Machines (SVM) ML algorithm [16]. Briefly, the SVM is trained on a dataset of known DTIs and learns the optimal hyperplane that separates the (m,p) pairs that interact from those that do not. While SVM can use vector representations of the data (i.e., descriptors for proteins and molecules), thanks to the so-called “kernel trick” [17], they can also find this hyperplane based on particular similarity measures between (m,p) pairs of training dataset, and called kernel functions *K*, without requiring explicit representation of the data.

A general method to build a kernel on (m,p) pairs is to use the Kronecker product of molecule and protein kernels [18]. Given a molecule kernel $K_{molecule}$ and a protein kernel $K_{protein}$, the Kronecker kernel $K_{pair}$ is defined by:

$$K_{pair}\left(\left(m,p\right),\left(m^{\prime },p^{\prime }\right)\right)=K_{molecule}\left(m,m^{\prime }\right)\times K_{protein}\left(p,p^{\prime }\right)$$

For proteins, we used a centered and normalized Local Alignment kernel (*LAkernel*), which mimics the Smith–Waterman alignment score between two proteins [19]. For the molecules, we used a centered and Tanimoto kernel, that uses molecular descriptors based on the number of fragments of a given length on the molecular graph [20].

The *LAkernel* has three hyperparameters: the penalties for opening (*o*) and extending (*e*) a gap, and the β parameter which controls the contribution of non-optimal local alignments to the final score. The Tanimoto kernel has one hyperparameter: the length *d* of the paths up to which paths on the molecule structure are considered. According to [11], we used the following values for these hyperparameters: o=20, e=1, and β=1 for the *LAkernel*, and d=14 for the Tanimoto kernel. The SVM also requires a regularisation parameter classically called *C*, which controls the trade-off between maximising the margin (i.e., the distance separating the hyperplane and the two classes distributions) and minimizing classification error on the training points. This parameter was set to C=10 for both RN- and BN-datasets, based on the nested cross-validation (CV) scheme, as described in Section 2.3.

SVM is a kernel-based ML algorithm. In the context of chemogenomics, it relies on similarity (or kernel) matrices between (m,p) pairs. Other algorithms, such as RF, are feature-based, and rely on explicit descriptors of proteins and ligands. To compare the performance of the kernel-based SVM to a feature-based approach, we compared our SVM to a RF on the RN-datasets. For the RF algorithm, we considered Extended-Connectivity Fingerprints (ECFP) [21] as molecular descriptors, and 1920-dimensional feature vectors summarizing physicochemical properties as protein descriptors, as in [22]. We considered four hyperparameters for RF: the number of trees; the minimum number of samples required at a leaf node; the minimum number of samples required to split an internal node; and the maximum depth of a tree. These hyperparameters were optimized based on a nested cross-validation scheme, as described in Section 2.3.

### 2.3. Performance Evaluation and Hyperparameters Optimisation

We used a nested cross-validation (CV), which allows to combine model selection and model evaluation without overfitting the dataset, as classically observed with a simple CV scheme [23,24]. In the nested CV scheme, the CV procedure for hyperparameter optimization (called “the inner CV”) is nested inside the CV procedure for performance evaluation (called “the outer CV”). The dataset is split into N folds: in each outer split, one fold is separated to form a test set. The N-1 remaining folds define an inner split. The hyperparameters are optimized on this inner split, based on a simple CV scheme. The set of hyperparameters providing the best inner CV prediction performance is then used on the test set of the corresponding outer split to evaluate the prediction scores. Thus, the model is tuned on the inner split, and performance of the model is evaluated on the test set of the outer split that was never used for model tuning. This procedure is repeated N times for each of the N outer splits, providing a mean and a variance for the performance scores. Figure S1 in Supplementary file presents a workflow chart describing a 5-fold nested CV used in the present study.

We used the following scores to quantify prediction performance of the classifiers:

- the Area Under the Receiver Operating Characteristic curve (ROC-AUC) [25]. The ROC curve represents true positive rate as a function of false positive rate, for all thresholds on the prediction score. Intuitively, the ROC-AUC score can be interpreted as the probability that the classifier assigns a higher score to a positive interaction than to a negative interaction.
- the Area Under the Precision-Recall curve (AUPR) [26], which indicates how far the scores of true positives are from those of true negatives, on average;
- the Recall, representing the fraction of positive examples that are retrieved;
- the Precision, representing the fraction of true positives retrieved among predicted positives;
- the False Positive Rate (FPR), representing the fraction of true negatives among predicted positives.

More precisely, we used a N=5 fold nested CV scheme to select the hyperparameter C of the SVM algorithm: RN-datasets (or BN-datasets) are split into *N* = 5 folds. Each fold comprises the same number of positive and negative DTIs. For the BN datasets, all molecules and all proteins appear in the same number of positive and negative DTIs, in each fold, as described in Section 2.1. Among the values {0.1, 1, 10, 100, 1000}, *C* = 10 consistently leads to the best performance across folds, both in terms of ROC-AUC and AUPR, and both on the RN- and BN-datasets.

We used the same nested CV scheme to optimize the hyperparameters of the RF algorithm (listed in Section 2.2) and to evaluate its performance on the RN-datasets. The number of trees was selected to be 600, chosen from {200, 400, 600}; the minimum number of samples required to be at a leaf node was selected to be 1, chosen from {1, 2, 5, 10}; the minimum number of samples required to split an internal node was selected to be 5, chosen from {2, 5}; and the maximum depth of the tree was selected to be 20, chosen from {10, 20}. The prediction scores were determined as for the SVM algorithm.

### 2.4. Flowcharts of DTI Prediction and Target Identification

**Figure 2:**
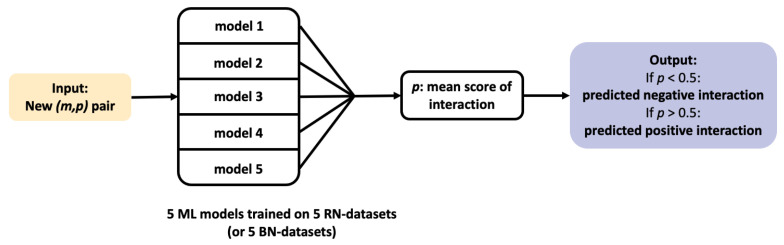
Flowchart of the Drug-Target Interaction (DTI) prediction pipeline.

**Figure 3:**
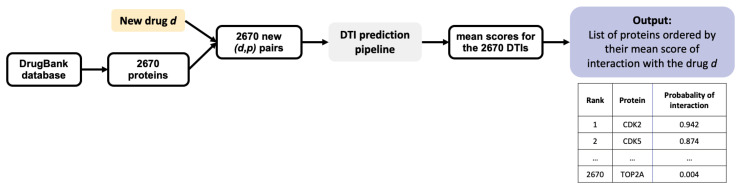
Flowchart of the target identification pipeline.

In the present paper, we discuss two types of problems that we solve using ML algorithms: first, the prediction of new pairs (m,p) of interacting molecules and proteins, which we call DTI (Drug-Target Interaction) prediction, and second, the identification of new targets for a given drug. The former is only discussed in Section 3.1, where DTI prediction is used to evaluate the overall prediction capabilities of ML algorithms, and to determine the distribution of the prediction scores of positive and negative DTI, respectively. We used these distributions to determine thresholds for the latter problem, i.e., target identification for a given drug, which is the central topic of the paper. Figure 2 illustrates the pipeline for DTI prediction: 5 ML models are trained on 5 RN-datasets (or 5 BN-datasets), providing 5 interaction scores for each new (m,p) pair. These 5 scores are averaged to provide a final score. Figure 3 illustrates the pipeline for target identification: for each new drug *d*, 2670 (d,p) pairs are formed between this drug and each of the 2670 proteins *p* present in the DB-Database. DTI prediction is performed for each pair, as described above and illustrated on Figure 2. This provides a mean score of interaction with this drug for each of the 2670 proteins, which are then ranked accordingly. The candidate targets for this drug are the top ranked proteins with a score above a given threshold.

## 3. Results

### 3.1. Performance of the SVM and RF Algorithms on the RN-Datasets

In the present paper, we focus on using ML chemogenomics approaches to identify target candidates for phenotypic hit molecules. The first step is to train the ML algorithms. More precisely, training a ML chemogenomics algorithm from a large DTIs database is an example of Positive-Unlabelled (PU) learning problem. Indeed, in practice, most databases only contain positive examples (that is to say, known DTIs), while all other possible interactions between molecules and proteins present in the data are unlabeled, whether because they have never been tested, or because they are negative interactions that have not been published or included in the database. Most of the unlabeled interactions are usually considered as true negatives. Therefore, in chemogenomics, the classical approach is to label as negatives a randomly chosen subset of the unlabeled interactions. This allows to convert a PU learning problem a into Positive-Negative (PN) learning problem for which many efficient ML algorithms are available.

We considered a ML algorithm based on SVM, with the *LAkernel* [27] and the Tanimoto kernel [20] for proteins and molecules, respectively, because these methods displayed good prediction performances in previous chemogenomic studies, on average [11,28,29]. The *LAkernel* is related to the Smith–Waterman score [19], but while the latter only keeps the contribution of the best local alignment between two sequences to quantify their similarity, the *LAkernel* sums up the contributions of all possible local alignments, which proved to be efficient for detecting remote homology.

While the purpose of this paper is not to discuss the choice of the ML algorithm, but rather to study how best to train it for the particular task of target identification, we also include a comparison of the SVM with a feature-based ML algorithm, i.e., Random Forests (RF) [30,31].

The two algorithms were trained on the 5 RN-datasets described in Section 2.1, using a 5-fold nested cross-validation scheme, as detailed in Section 2.2 and Section 2.3. A threshold of 0.5 on the output score was chosen to discriminate between positive and negative predictions.

Table 1 shows the mean performance scores of the SVM and RF algorithms, when cross-validated on the RN-datasets. In the context of target identification, it is important to limit the FPR, to avoid unnecessary experimental validation. However, a threshold of 0.5 over the probability scores was used to separate predicted positive interactions from predicted negative interactions, as classically, although in practical cases, a higher threshold would be chosen to select target candidates, in order to reduce the number of experimental tests to the predictions with the highest confidence. The results in Table 1 show that the SVM clearly outperforms RF across all performance scores, including FPR. We therefore retained the SVM for the rest of the paper.

**Figure 4:**
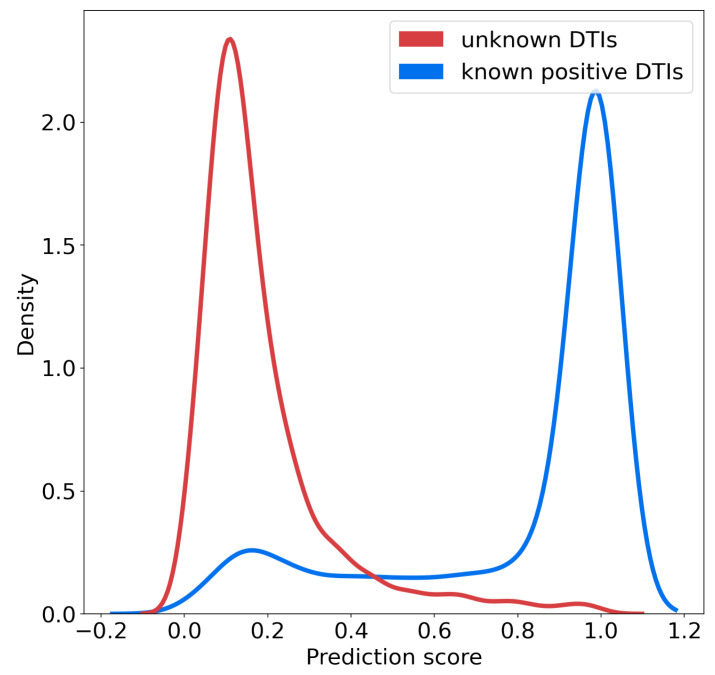
Distribution of the probability scores predicted for known positive DTIs and randomly chosen negative DTIs among unlabeled DTIs.

We studied the distributions of the probability scores for positive and unlabeled (presumably, mainly negative) interactions for the SVM algorithm, according to the nested CV scheme. Figure 4 shows that these two distributions are well separated, and also suggests that on the RN-dataset, a threshold of 0.7 over the prediction score can be used to predict positive interactions with high confidence. In addition, the rank of a predicted interaction is also an important criterion to consider, because the goal of virtual screens is to drastically reduce the number of experiments to perform. When the goal is to identify hit molecules against a given therapeutic target, typically, the top 5% percent of the best-ranked molecules are screened [32]. Usually, an experimental assay with a simple readout has been set up for the target of interest, which allows to evaluate relatively high numbers of candidate molecules selected in the virtual screen. The inverse problem of target identification is more difficult because validation requires to test the phenotypic hit molecule in a different biological assay for each predicted target considered for experimental evaluation. This obviously requires much more time and effort, because these assays may not all be available, and therefore, may have to be designed. This can be a real challenge if the function of a candidate target is not suitable to design a simple biological test. Therefore, we added the stringent but realistic threshold of top 1% in rank. In other words, in the following, we will consider as candidate targets proteins with a predicted score above 0.7 and ranked among the top 1% of the tested proteins, to simulate a realistic experimental setting. We discuss how to best train the algorithm in order to minimize the number of useless biological experiments that would be undertaken for false positive targets satisfying these two criteria, because this represents a real bottleneck for real-case studies. Consequently, in what follows, since the DB-Database comprises 2 670 proteins, we will consider as candidate targets only proteins with a probability score above 0.7 and rank smaller than or equal to 27.

### 3.2. Statistical Analysis of the DrugBank Database

**Figure 5:**
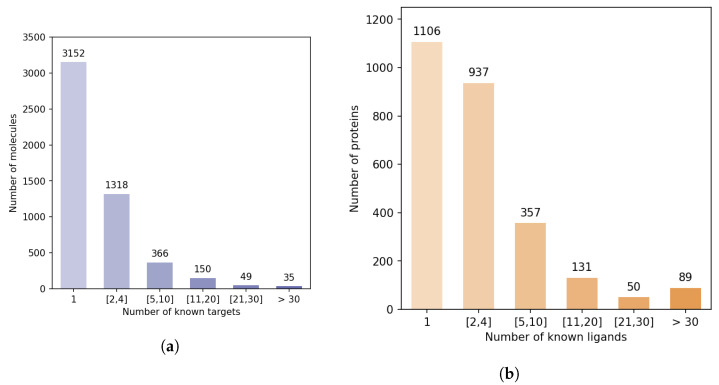
Statistical bias in the DB-Database. (**a**) Distribution of the molecules according to their number of targets in the DB-Database. (**b**) Distribution of the proteins according to their number of ligands in the DB-Database.

The DrugBank database [15] is a widely used bio-activity database. While much smaller than PubChem or ChEMBL, it provides high-quality information for approved and experimental drugs along with their targets. It contains around 15,000 curated DTIs involving 2670 human proteins (this set of proteins can be viewed as the “druggable” human proteome), and 5070 druglike molecules, corresponding to the DB-Database described in Section 2.1. This database is relevant for training of ML models for DTI predictions involving human proteins and drug-like molecules. However, Figure 5 shows that there is a strong discrepancy between the number of known ligands per protein, or known protein targets per molecule.

Indeed, the majority of proteins have 4 or fewer known ligands, while around 140 proteins have more than 21 ligands. We defined categories of proteins, depending on their number of known ligands (1, 2 to 4, 5 to 10, 11 to 20, 21 to 30, more than 30), and calculated the number of DTIs in the DB-Database in each category. Overall, according to Table 2, 5.2% of the proteins are involved in 44% of DB-Database DTIs.

This bias arises from the fact that a few diseases like cancer or inflammatory diseases have attracted most research efforts, and many ligands have been identified against related therapeutic targets, compared to other less studied human proteins. For example, Prostaglandin G/H synthase 2, a well-known protein involved in inflammation, has 109 drugs reported at DrugBank. This statistical bias affects training of the SVM and is expected to perturb identification of targets for hit molecules, potentially by enriching top ranked proteins in false positive targets that have many known ligands.

### 3.3. Examples Illustrating the Impact of Learning Bias for Target Identification

Once trained, a ML algorithm identifies targets for a hit molecule by providing a list of proteins ranked by decreasing order of the estimated probability score of all (protein, hit) pairs. Candidate targets are chosen based on their probability score, their rank, and on potential prior biological knowledge that would highlight their relation to the considered disease. For example, a top ranked protein involved in cell cycle would be considered as a realistic candidate target for a hit identified in a cell proliferation screen in cancer research. The presence of many false positive targets among the top ranked proteins will not only lead to undertake useless experiments, but also potentially to discard true predicted targets pushed further down the list. Let us illustrate this problem in the case of 3 molecules, randomly chosen among marketed drugs with only one known target in DrugBank. Assuming that their targets have been well characterized because they are marketed molecules, most of the other top ranked predicted targets will be false positive predictions. The 3 considered molecules are: alectinib (DrugBank ID DB11363, target: ALK), lasmiditan (DrugBank ID DB11732, target: HTR1F), and doxapram also known as angiotensin II (DrugBank ID DB11842, target: AGTR1). We orphanized these 3 molecules (i.e., we suppressed their single known target from the train set), as if they were hits from phenotypic screens, and used the SVM algorithm presented in Section 2.2 on the RN-datasets to predict their targets. For each molecule, the results consist in a list of the 2670 proteins in the DB-Database, ranked by decreasing order of score.

As shown in the RN-datasets columns of Table 3, none of the known targets for those drugs are among the candidate targets as defined in Section 3.1. More precisely, for DB11363 and DB11842, although the probability scores of their known targets are above 0.7 (values of 0.8 and 0.76 respectively), their rank is 31 in both cases, above the threshold of 27. For DB11732, the probability score of HTR1F is 0.67, with a rank of 107, and HTR1F would not either have been classified among the candidate targets for testing.

Analysis of the results highlighted that some of the best ranked candidate targets are frequent targets. For example, prothrombin F2 (120 ligands), cyclin dependant kisase CDK2 (137 ligands), and dopamine receptor 2 DRD2 (109 ligands) are top ranked predicted targets respectively for DB11842 (score of 0.97, rank 2), DB11732 (score 0.98, rank 1) and DB11363 (score 0.94, rank 5). The three ranked lists are provided in full in the github repository mentioned under “Data Availability Statement”.

These examples illustrate the impact of false positive predictions for target identification, because they can lead to discard even high-scoring true targets as for DB11363 and DB11842.

### 3.4. Choice of Negative Examples to Correct Statistical Bias

**Figure 6:**
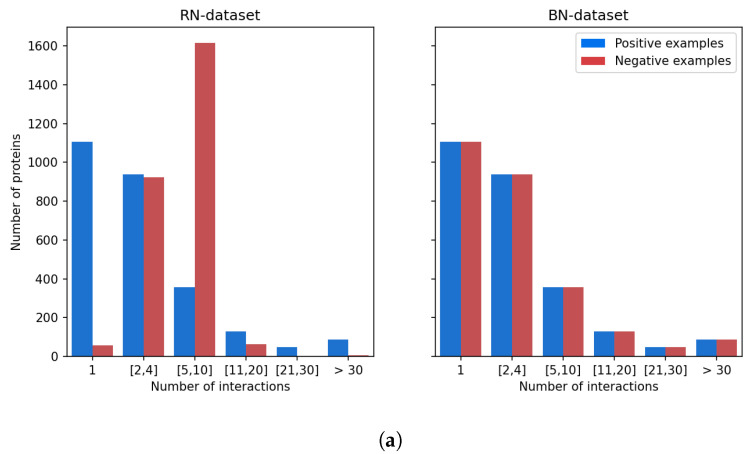

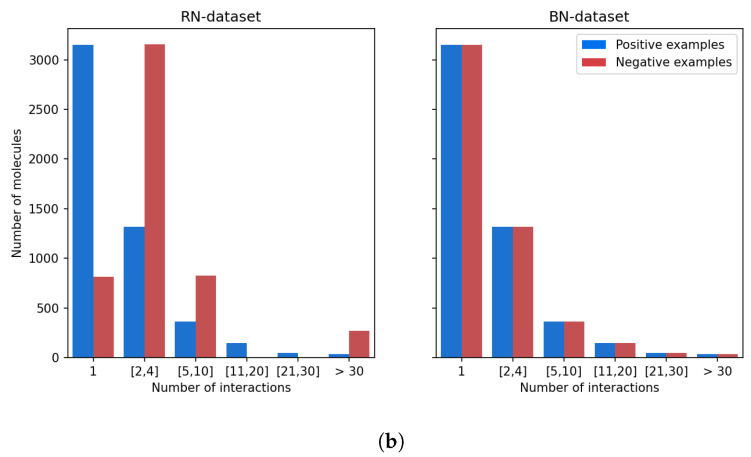
Balancing the BN-datasets. (**a**) Distribution of the proteins according to the number of positive examples or negative examples in which they are involved. (**b**) Distribution of the molecules according to the number of positive examples or negative examples in which they are involved.

Our observation that high-scoring false positives tend to have a large number of known ligands led us to make the assumption that the model trained using randomly sampled negative interactions is biased towards proteins with many known ligands, as well as possibly drugs with many known targets. This suggested us to choose negative DTIs in such a way that the training dataset contains, for each molecule and for each protein, as many positive than negative DTIs. The corresponding so-called BN-datasets (for Balanced Negatives-datasets) are detailed in Section 2.1. Note that what we mean by “balanced” in the BN-dataset is that negative examples present the same bias as the positive examples: all molecules and all proteins appear in the same number of positive and negative DTIs. As shown in Figure 6: (1) in the positive examples, the distribution of known protein targets per molecule is similar to that of proteins known (chosen, in fact) not to interact per molecule in the negative examples; (2) in the positive examples, the distribution of known ligands per protein is similar to that of molecules known (chosen, in fact) not to interact per protein in the negative examples. This prevents proteins with many known ligands to be viewed by the algorithm as statistically much more probable targets, leading to many false positive predictions among this category of proteins. We recall that the BN-datasets contains the same positive DTIs as the RN-datasets, the former differing from the latter only by the negative DTIs.

The SVM algorithm presented in Section 2.2 was trained on the BN-datasets. As discussed above, for the problem of target identification, reducing the number of false positives among the top-ranked proteins is critical. Table 4 reports, for prediction score thresholds of 0.5 (usually considered) and 0.7 (considered in the present paper), the cross-validated FPR scores on these two training sets. It shows a strong statistical bias in FPR for the RN-datasets between proteins with few or with many known ligands, and it illustrates that training on the BN-datasets greatly reduced this bias.

To highlight the impact of this bias correction in terms of target prediction, we show in Table 3 the prediction results for the 3 molecules discussed in Section 3.3, when the algorithm is trained with the RN-datasets or with the BN-datasets. When trained on the RN-datasets, none of the true targets would have been considered as a positive candidate target for testing, because of a score below 0.7 or a rank above 27, as discussed above. Training on the BN-datasets greatly improved the ranks and scores of the three true targets, and reduced the number of false positives, allowing the 3 corresponding true targets to fulfill the rank and score criteria defined in Section 3.1 to become candidate target for testing.

To better illustrate the interest of the proposed scheme for the choice of negative DTIs on a larger number of drugs we considered the 200-positive-dataset consisting of 200 DTIs involving 200 marketed drugs with 4 of less known targets, as described in Section 2.1. This “difficult” test set was chosen because the aim was to mimic newly identified phenotypic hits, for which known targets are expected to be scarce. For each drug, we artificially reproduced the process of target identification: the corresponding DTI was removed from the train set, a new SVM classifier was trained and used to score 2670 DTIs involving this drug and all proteins of the DB-Database. We compared the top-ranked predicted targets obtained when the algorithm is trained on the RN-datasets versus on the BN-datasets, as well as the number of removed false positive DTIs that would have been retrieved as candidates for testing (i.e., with a score above 0.7 and a rank lower or equal to 27).

Overall, training with the BN-datasets improved the predictions: the number of false positive DTIs decreased for 106 drugs, remained unchanged in 85 drugs, and increased in 9 drugs, as compared to training with the RN-datasets. In particular, this improvement allowed one additional true positive interaction to reach a score above 0.7 and a rank below 27: 104 true targets were retrieved as candidates when training with BN-datasets, compared to 103 when training with RN-datasets. For the corresponding 104 drugs, the number of false positives decreased by 2.9 in average, and the rank of the true interactions decreased by 1.8 in average, bringing them even closer to the top ranked predicted proteins, and more likely to be chosen for experimental validation. Consistent with the results in Section 3.3 for the 3 example molecules, on average over the 200 considered molecules, the number of useless experiments potentially undertaken would have decreased when training with the BN-datasets.

We also made predictions for the 200 negative DTIs of the corresponding 200-negative-dataset, involving the same molecules as the 200-positive-dataset. Predictions were made by the classifier trained on the RN- or BN- datasets. Overall the distributions of the prediction scores were very similar in both cases, centered around 0.2, and similar to that shown for the RN-dataset in Figure 4. Among the 200 negative pairs, only 2 pairs were predicted as positives, for the two RN- and BN- datasets. This can be viewed as a sanity check indicating that the proposed method did not introduce bias in the prediction of negative DTIs, while it globally improved the predictions of positive DTIs.

## 4. Discussion

The goal of the present paper was to tackle the question of protein target identification for new drug candidates, using ML-based chemogenomics. Indeed, these approaches can be run at a large scale in the protein space, including in their scope proteins with no known 3D structures, or proteins for which few, or even no ligands are known. Another key asset is that they can be applied to drugs with few, or even no known targets, as illustrated on the 200-positive-dataset. This is of particular importance because new phenotypic drugs are often orphan (i.e., have no known protein target) when they are identified. No other computational method presents these advantages. However, before making predictions, ML chemogenomic algorithms need to be trained on a database of known DTIs, which raises a few issues.

First, these databases are biased in terms of the number of protein targets per molecule, or of ligand molecules per protein, as shown for the DrugBank database used in our study. While we are aware that other and larger DTIs databases could have been used, the purpose of our study was not to discuss the choice of training set, in particular because other databases will also present the same type of bias as the DrugBank, for the same reasons. This point is rarely discussed in ML chemogenomic studies.

Second, the performance of ML algorithms in chemogenomics are usually evaluated based on AUPR and ROC-AUC scores in cross-validation procedures. However, the identification of true protein targets for phenotypic hit molecules in real case studies may become a challenge when the algorithm is trained on a biased dataset. Indeed, despite very high AUPR and ROC-AUC scores, false positive targets can be found among top-ranked proteins, and correspond to proteins with many known ligands. In target identification studies, biological experiments are guided by the predicted scores and ranks of candidate proteins. Training on a biased dataset may lead not only to conduct useless experiments, but also to discard true positive targets because their scores are below the considered threshold, or because their rank is too high due to the presence of false positives among the top-ranked proteins. This point is also rarely discussed in ML chemogenomic studies, usually focusing on cross-validation schemes that does not correspond to real case applications.

Third, training databases such as the DrugBank only contain positive examples, and therefore, negative examples are usually randomly chosen among unlabeled DTIs in order to train the ML algorithms. It is however unclear that this is an optimal choice for target identification.

The key result of the present paper was to show that choosing an equal number of positive and negative DTIs per molecule and per protein helps decrease the FPR in biased datasets, and improves the identification of true targets for a given drug. Three striking examples are given for the case study of three drugs (DB11363, DB11842, and DB11732) that were “orphanized” (all their known DTIs were removed from the training set) to illustrate the most difficult situation encountered in the case of new phenotypic drugs: training with the BN-datasets allowed to recover the true target in all cases, while none of them would have been retrieved when training with the RN-datasets. To illustrate the advantage of the proposed scheme for the choice of negative interactions, we used a threshold of 0.7 over the probability scores to identify candidate targets for experimental testing, although proteins with scores above 0.5 are classified as positives. This threshold of 0.7 was guided by the results in Figure 4, in order to select highly probable positive targets. It can be adjusted to a different value if the algorithm is trained with other databases, whether through the same kind of plot, or through a ROC-curve in order to correspond to a predefined false positive rate.

We added the stringent threshold of 1% on the ranks of proteins to define which targets would be tested. This threshold could also be adjusted depending on available resources for experimental validation. The issue we identified and addressed in this paper does not depend on the scores and rank thresholds used, and choosing equal numbers of positive and negative DTIs per molecule and per protein for the training set will limit the number of false positives independently of the choice of thresholds, as shown in Table 4 in the case of the threshold on the prediction score. Finally, while the proposed scheme for the choice of negative examples was presented here in the context of target identification for hit molecules, it is of general interest and should be applicable to other types of PU learning problems when bias is present in the training set, which is a very common situation, in particular in many biological databases.

## Figures and Tables

**Table 1:** Performance of the SVM and RF algorithms for DTI predictions on the RN-datasets.

Algorithm	AUPR	ROC-AUC	Recall	Precision	FPR
**SVM**	85.5±0.2	88.0±0.1	82.0±0.4	93.3±0.4	5.9±0.4
**RF**	73.5±0.8	79.1±0.7	76.8±1.0	80.6±0.8	18.5±1.0

**Table 2:** Distribution in the DB-Database of the number of DTIs involving proteins from various categories, according to their number on known ligands.

Protein nb of Ligands	nb of Interactions
1	1106
2 to 4	2527
5 to 10	2404
11 to 20	1920
21 to 30	1238
>30	5442

**Table 3:** DTI prediction results for 3 marketed drugs, when the algorithm is trained on the RN-datasets or the BN-datasets: number of False Positive predicted targets, score and rank of the true target.

	RN-Datasets			BN-Datasets		
**Drug**	**FP**	**Target Score**	**Target Rank**	**FP**	**Target Score**	**Target Rank**
DB11363	27	0.8	31	16	0.8	3
DB11842	27	0.76	31	26	0.85	18
DB11732	27	0.67	107	26	0.83	17

**Table 4:** Rate of false positives for proteins with various numbers of known ligands.

	FPR (Threshold = 0.5)		FPR (Threshold = 0.7)	
Prot in Category	RN-Datasets	BN-Datasets	RN-Datasets	BN-Datasets
0	2.2±0.4	3.1±0.5	0.5±0.4	0.7±0.5
1	3.7±0.5	3.1±0.8	1.5±0.1	1.1±0.7
2 to 4	5.1±0.9	6.4±0.8	2.4±0.8	2.2±0.8
5 to 10	9.9±0.9	8.3±0.6	4.4±0.9	3.3±0.5
11 to 20	13.8±1.7	10.6±0.5	7.3±1.9	3.9±1.1
21 to 30	23.0±4.9	12.0±3.0	11.4±2.7	5.6±2.0
>30	18.6±2.8	9.0±0.4	11.0±2.1	4.5±0.3

## Data Availability

Datasets and results, presented in this study, are available at https://github.com/njmmatthieu/dti_negative_examples_data.git, included a README.md file describing them.

## Supplementary Materials

The following are available online at https://www.mdpi.com/article/10.3390/ijms22105118/s1, Figure S1: Nested Cross Validation Workflow with N = 5 outer splits.

## Abbreviations

The following abbreviations are used in this manuscript:

BN-dataset	Balanced Negatives-dataset
DB-Database	DrugBank Database
CV	Cross-Validation
DTI	Drug-Target Interaction
FP	False Positive
FPR	False Positive Rate
(*m,p*)	(molecule, protein)
LAkernel	Local Alignement kernel
ML	Machine Learning
PU learning	Positive-Unlabelled learning
RF	Random Forests (algorithm)
ROC-AUC	Area Under the Receiver Operating Characteristic curve
RN-dataset	Random Negatives-dataset
SVM	Support Vector Machine (algorithm)

## References

[1] Swinney D.C., Anthony J. (2011). How were new medicines discovered?. Nat. Rev. Drug Discov..

[2] Moffat J.G., Vincent F., Lee J.A., Eder J., Prunotto M. (2017). Opportunities and challenges in phenotypic drug discovery: An industry perspective. Nat. Rev. Drug Discov..

[3] Martinez-Lopez Y., Caballero Y., Barigye S.J., Marrero-Ponce Y., Millan-Cabrera R., Madera J., Torrens F., Castillo-Garit J.A. (2017). State of the Art Review and Report of New Tool for Drug Discovery. Curr. Top. Med. Chem..

[4] Xu X., Huang M., Zou X. (2018). Docking-based inverse virtual screening: Methods, applications, and challenges. Biophys. Rep..

[5] Vert J.P., Jacob L. (2008). Machine Learning for In Silico Virtual Screening and Chemical Genomics: New Strategies. Comb. Chem. High Throughput Screen..

[6] Bolton E.E., Wang Y., Thiessen P.A., Bryant S.H. (2008). PubChem: Integrated Platform of Small Molecules and Biological Activities. Annual Reports in Computational Chemistry.

[7] Jacob L., Vert J.P. (2008). Protein-ligand interaction prediction: An improved chemogenomics approach. Bioinformatics.

[8] Liu Y., Wu M., Miao C., Zhao P., Li X.L. (2016). Neighborhood Regularized Logistic Matrix Factorization for Drug-Target Interaction Prediction. PLoS Comput. Biol..

[9] Svetnik V., Liaw A., Tong C., Culberson J.C., Sheridan R.P., Feuston B.P. (2003). Random Forest: A Classification and Regression Tool for Compound Classification and QSAR Modeling. J. Chem. Inf. Comput. Sci..

[10] Yamanishi Y., Pauwels E., Saigo H., Stoven V. (2011). Extracting Sets of Chemical Substructures and Protein Domains Governing Drug-Target Interactions. J. Chem. Inf. Model..

[11] Playe B., Azencott C.A., Stoven V. (2018). Efficient multi-task chemogenomics for drug specificity prediction. PLoS ONE.

[12] Playe B., Stoven V. (2020). Evaluation of deep and shallow learning methods in chemogenomics for the prediction of drugs specificity. J. Cheminform..

[13] Pahikkala T., Airola A., Pietilä S., Shakyawar S., Szwajda A., Tang J., Aittokallio T. (2015). Toward more realistic drug-target interaction predictions. Briefings Bioinform..

[14] Lipinski C.A., Lombardo F., Dominy B.W., Feeney P.J. (2001). Experimental and computational approaches to estimate solubility and permeability in drug discovery and development settings 1PII of original. Adv. Drug Deliv. Rev..

[15] Law V., Knox C., Djoumbou Y., Jewison T., Guo A.C., Liu Y., Maciejewski A., Arndt D., Wilson M., Neveu V. (2014). DrugBank 4.0: Shedding new light on drug metabolism. Nucleic Acids Res..

[16] Cortes C., Vapnik V. (1995). Support-vector networks. Mach. Learn..

[17] Schölkopf B., Tsuda K., Vert J.P. (2004). Kernel Methods in Computational Biology.

[18] Erhan D., L’Heureux P.J., Yue S.Y., Bengio Y. (2006). Collaborative Filtering on a Family of Biological Targets. J. Chem. Inf. Model..

[19] Smith T., Waterman M. (1981). Identification of common molecular subsequences. J. Mol. Biol..

[20] Swamidass S.J., Chen J., Bruand J., Phung P., Ralaivola L., Baldi P. (2005). Kernels for small molecules and the prediction of mutagenicity, toxicity and anti-cancer activity. Bioinformatics.

[21] Rogers D., Hahn M. (2010). Extended-Connectivity Fingerprints. J. Chem. Inf. Model..

[22] Ong S.A., Lin H.H., Chen Y.Z., Li Z.R., Cao Z. (2007). Efficacy of different protein descriptors in predicting protein functional families. BMC Bioinform..

[23] Hastie T., Tibshirani R., Friedman J. (2009). The Elements of Statistical Learning.

[24] Cawley G.C., Talbot N.L. (2010). On Over-fitting in Model Selection and Subsequent Selection Bias in Performance Evaluation. J. Mach. Learn. Res..

[25] Hanley J.A., McNeil B.J. (1982). The meaning and use of the area under a receiver operating characteristic (ROC) curve. Radiology.

[26] Raghavan V., Bollmann P., Jung G.S. (1989). A critical investigation of recall and precision as measures of retrieval system performance. ACM Trans. Inf. Syst..

[27] Saigo H., Vert J.P., Ueda N., Akutsu T. (2004). Protein homology detection using string alignment kernels. Bioinformatics.

[28] Wang Y.C., Zhang C.H., Deng N.Y., Wang Y. (2011). Kernel-based data fusion improves the drug–protein interaction prediction. Comput. Biol. Chem..

[29] Meslamani J., Rognan D. (2011). Enhancing the Accuracy of Chemogenomic Models with a Three-Dimensional Binding Site Kernel. J. Chem. Inf. Model..

[30] Cao D.S., Zhang L.X., Tan G.S., Xiang Z., Zeng W.B., Xu Q.S., Chen A.F. (2014). Computational Prediction of Drug—Target Interactions Using Chemical, Biological, and Network Features. Mol. Inform..

[31] Breiman L. (2001). Random Forests. Mach. Learn..

[32] Adeshina Y.O., Deeds E.J., Karanicolas J. (2020). Machine learning classification can reduce false positives in structure-based virtual screening. Proc. Natl. Acad. Sci. USA.

