# Supplementary material for: Drug Target Identification with Machine Learning: How to Choose Negative Examples
Source: Int J Mol Sci. 2021 May 12;22(10):5118. doi: 10.3390/ijms22105118 (PMC8151112; doi:10.3390/ijms22105118)
Supplement: Supplementary file 1 [file ijms-22-05118-s001.zip › ijms-1182453-supplementary.pdf]

Supplementary Materials:

1. Flowchart of nested Cross Validation

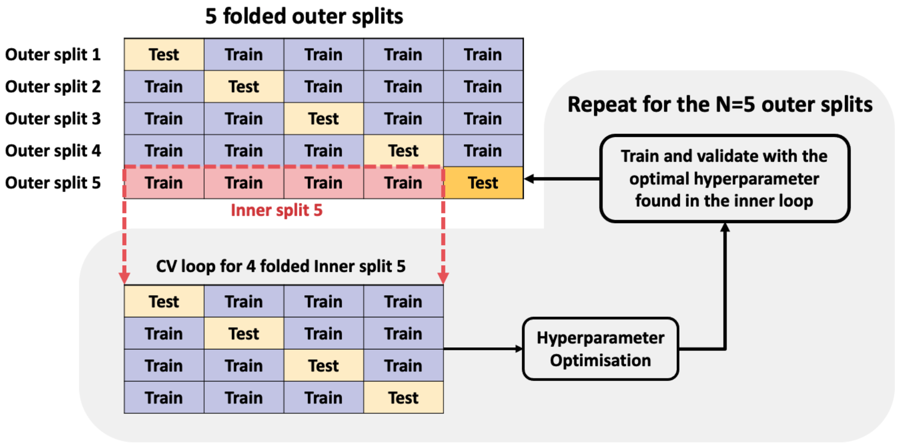

Figure S1. Nested Cross Validation Workflow with N=5 outer splits.
